# Dung beetle vicariant speciation in the mountains of Oaxaca, Mexico, with a description of a new species of *Phanaeus* (Coleoptera, Geotrupidae, Scarabaeidae)

**DOI:** 10.3897/zookeys.743.23029

**Published:** 2018-03-14

**Authors:** Bert Kohlmann, Alfonsina Arriaga-Jiménez, Matthias Rös

**Affiliations:** 1 Universidad EARTH, AP 4442-1000, San José, Costa Rica; 2 CIIDIR-Oaxaca, Instituto Politécnico Nacional, México; 3 CONACYT, CIIDIR-Oaxaca, Instituto Politécnico Nacional, México

**Keywords:** Biogeography, dry oak scrub-forest, last glacial maximum, *Phanaeus endymion* species group, *Phanaeus malyi*, revalidation, Sierra Norte, Sierra Sur, sister species, sky-islands dynamic

## Abstract

An analysis of vicariant speciation of *Geotrupes* and *Phanaeus* (Coleoptera, Geotrupidae, Scarabaeidae) from the mountains of Oaxaca, Mexico is undertaken. The new species of dung beetle (Coleoptera: Scarabaeidae) from Oaxaca, Mexico, *Phanaeus dionysius* **sp. n.** is described. Photos of the habitus and a distribution map are provided. *Phanaeus malyi* Arnaud is revalidated. An updated key for the *Phanaeus endymion* species group and new localities are also presented. An updated key for the *Geotrupes* of Oaxaca and new locality records are also submitted.

## Abstract (es)

Se realiza un análisis de especiación vicariante de *Geotrupes* y *Phanaeus* (Coleoptera, Geotrupidae, Scarabaeidae) de las montañas de Oaxaca, México. La siguiente especie nueva de escarabajo estercolero (Coleoptera: Scarabaeidae) se describe de Oaxaca: *Phanaeus dionysius* **sp. n.** Se incluyen fotografías, así como el respectivo mapa de distribución. Se revalida a la especie *Phanaeus malyi* Arnaud. Se presenta una clave puesta al día del grupo de especies de *Phanaeus endymion*, así como nuevos registros de distribución. Se incluye también una clave puesta al día para los *Geotrupes* de Oaxaca.

## Introduction

Recent collecting and taxonomic studies of dung beetles of the genera *Geotrupes* Latreille (Coleoptera: Geotrupidae) and *Phanaeus* MacLeay (Coleoptera: Scarabaeidae) in the mountains of Oaxaca have evidenced the existence of a repeated vicariant speciation pattern. This pattern seems to be located at present between the northern (Sierra Norte) and southern (Sierra Sur) mountain ranges of this state. An analysis of this vicariant speciation mechanism is presented, based on the great taxonomic similarity (sister species) shown by the *Geotrupes viridiobscurus* Jekel-*Geotrupes pecki* Howden and *Phanaeus dionysius* sp. n.-*Phanaeus zapotecus* Edmonds species pairs where the first species inhabits the northern and the second one the southern mountain systems.

In this paper Phanaeus (Notiophanaeus) dionysius sp. n. is also described, a species that inhabits dry oak scrub forest in the Mexican state of Oaxaca, between 1900 and 2200 m above sea level and belongs to the *Phanaeus endymion* species group. The number of *Phanaeus* in Mexico has been increasing during the last years. Since Edmonds and Zídek’s monographic analysis in 2012, three new species have been described just in 2017 from Mexico (Moctezuma and Halffter 2017, Moctezuma et al. 2017) and one more in 2018 from Ecuador (Arnaud 2018).

This new species brings the number of known Mexican *Phanaeus* species to 32 (see Edmonds and Zídek 2012, Moctezuma and Halffter 2017, Moctezuma et al. 2017). Mexico seems to be a hotbed for *Phanaeus* speciation because more than half of the species (32 out of 58 described species; 34 if we also include the USA) are distributed in this country. As a comparison, Colombia registers eight species (Medina et al. 2001), Costa Rica also eight species (Solís and Kohlmann 2012), and Panama only four (Solís and Kohlmann 2012). This suggests that *Phanaeus* has invaded North America, probably since the Miocene (Edmonds 1994), and that *Phanaeus* found empty niches (where there were no other Phanaeini genera competing) and/or reduced local competition that promoted speciation. We consider therefore that the most probable local North American ecological equivalent, the Oniticellini, were outcompeted by them or perhaps they were poorly represented from the beginning. Philips (2016) considers the invasion of North America by the Oniticellini from Asia via Beringia to be an old event, most likely taking place during the Eocene, or perhaps even earlier during the late Oligocene or early Miocene. On the mainland, the only species of *Attavicinus* Philips and Bell, and two species of *Liatongus* Reitter, are known at present, living in restricted niches like ant debris, carrion, and fungi (Philips 2016). Philips (2016) considers necrophagy and mycetophagy to have evolved recently, therefore suggesting that the Oniticellini could have changed their feeding habits recently under a competitive pressure process exerted by the Phanaeini.

An updated key as well as new localities and commentaries for species of the *Phanaeus endymion* species group are also presented. We revalidate *Phanaeus malyi* Arnaud, a member of the aforementioned species group. New distribution records for *Phanaeus endymion* species group (*Ph. bravoensis*, *Ph. halffterorum* and *Ph. huichol*) are included. New distribution records for *Geotrupes viridiobscurus* and *Geotrupes pecki* also are added (*G. pecki* was last reported 40 years ago; Howden 1974), and an analysis of their distribution is presented. An updated key for Oaxacan *Geotrupes* also is presented.

## Materials and methods

The superb personal collection of Julián Blackaller, in Soria, Guanajuato, Mexico, was reviewed; as well as the collection of the National Museum in Costa Rica and the Canadian Museum of Nature in Ottawa, Canada. Collections made by Arriaga-Jiménez as part of her studies on mountain dung-beetles, which are deposited in the entomology collection at the Institute of Ecology, Xalapa, Mexico, were also studied. Body measurements were made to the nearest 0.1 mm using an ocular micrometer with a Stemi DV4 stereoscope. Genital dissections and preparations were done following the techniques described by Zunino (1978). Genital structures were stored in microvials with glycerin.

The photos were taken by Alfonso Aceves from the Instituto de Ecología (INECOL), Xalapa, Mexico, using a Canon T2i camera, extension tubes, a 100 mm macro, and an external Canon flash. The photos of *Ph. malyi* and *Ph. pyrois* were taken by Ángel Solís from the Museo Nacional de Costa Rica using an Olympus OM-D E-M5 digital camera. Susana Guzmán-Gómez from the Instituto de Biología, UNAM, Mexico, took the photo of the pygidium of *Ph. zapotecus*, using a Zeiss AXIO Zoom V16 microscope, a Zeiss AxioCam MRc5 camera, and the ZEN (Zeiss Efficient Navigation) multifocal technology programme. The photos of *Ph. zapotecus* and the Suppl. material 1 (Figure S1) were taken by François Génier, from the Canadian Museum of Nature, Ottawa, using a Leica Z16 system and LAS software for image stacking.

The holotype, allotype, and two paratypes of *Ph. dionysius* are deposited in the Colección Entomológica (Entomology Collection), Instituto de Ecología, Xalapa, Mexico (IEXA). Further paratypes are deposited in: two paratypes (male and female) in the Canadian Museum of Nature, Ottawa (CMN); two paratypes (male and female) in the Julián Blackaller (JB) personal collection, Soria, Mexico; two paratypes (male and female) in the Patrick and Florent Arnaud (CPFA) personal collection, Saintry sur Seine, France; and two paratypes (male and female) in the Seção de Entomologia da Coleção Zoológica da Universidade Federal de Mato Grosso, Cuiabá (CEMT), Brasil.

## Taxonomy

### Phanaeus (Notiophanaeus) dionysius

Taxon classificationAnimaliaColeopteraScarabaeidae

Kohlmann, Arriaga-Jiménez & Rös sp. n.

http://zoobank.org/B4F5DBB5-6802-44F0-87FF-199895A96C80

**Figure 1.**
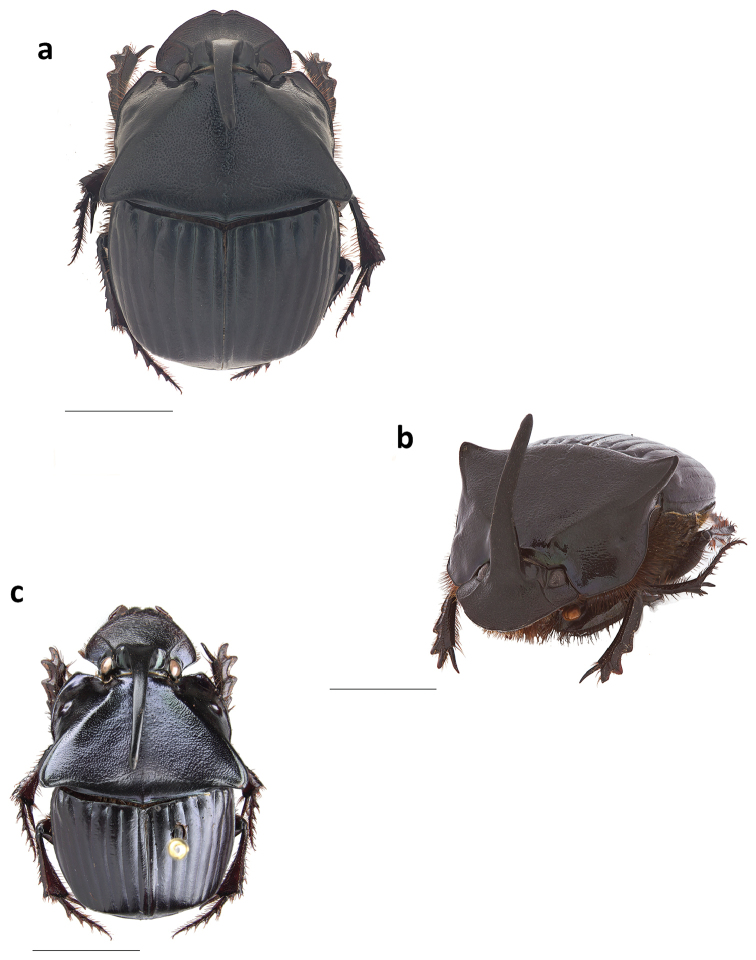
Major male habitus in **a** dorsal and **b** lateral view of *Phanaeus dionysius* sp. n., and **c** dorsal view of *Ph. zapotecus*. Scale bar= 5mm.

**Figure 2.**
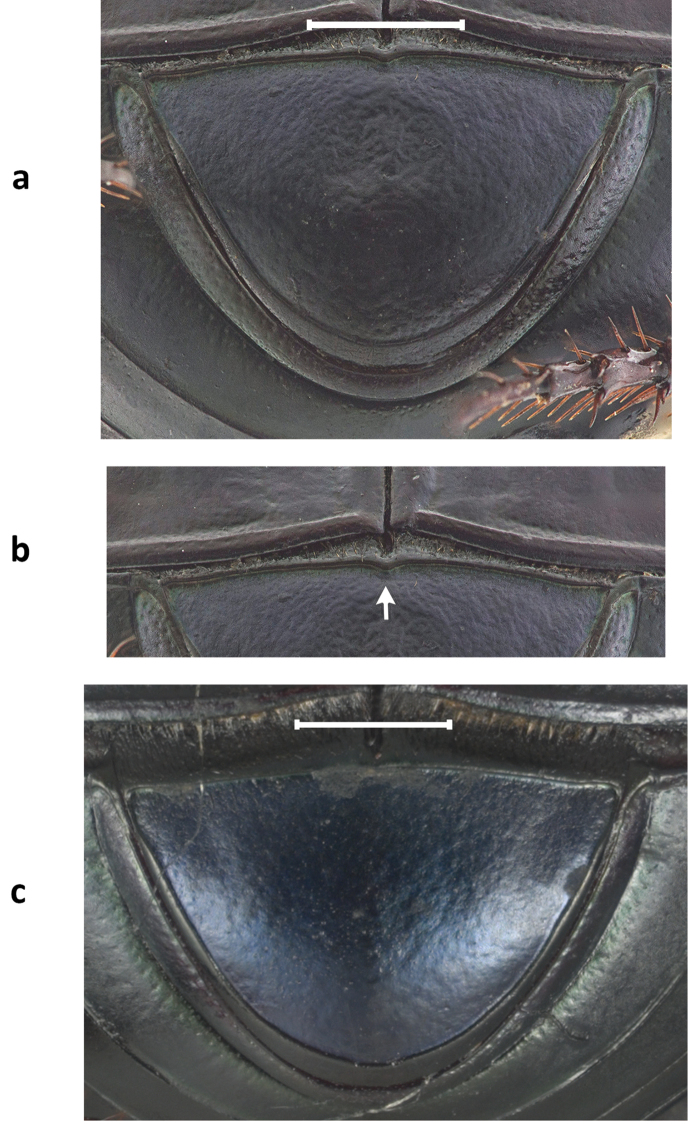
Pygidia of **a** *Ph. dyionysius* sp. n. **b** detail, arrow points to the triangle that forms the keel **c** *Ph. zapotecus*. Scale bar= 1mm.

**Figure 3.**
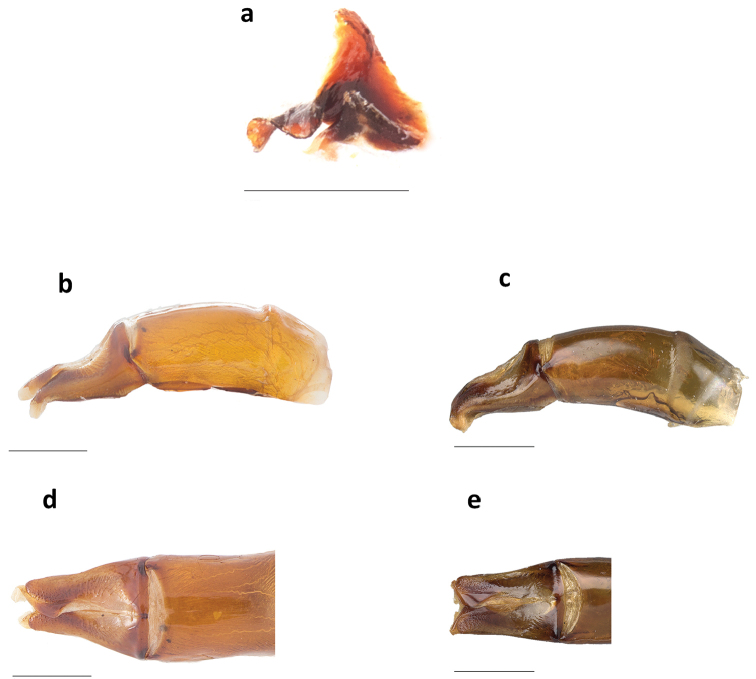
**a** Lamella copulatrix and **b** aedeagus of *Phanaeus dionysius* sp. n. **c** aedeagus of *Phanaeus zapotecus* **d** parameres of *Ph. dionysius* sp. n. **e** parameres of *Ph. zapotecus*. Scale bar= 1mm.

**Figure 4.**
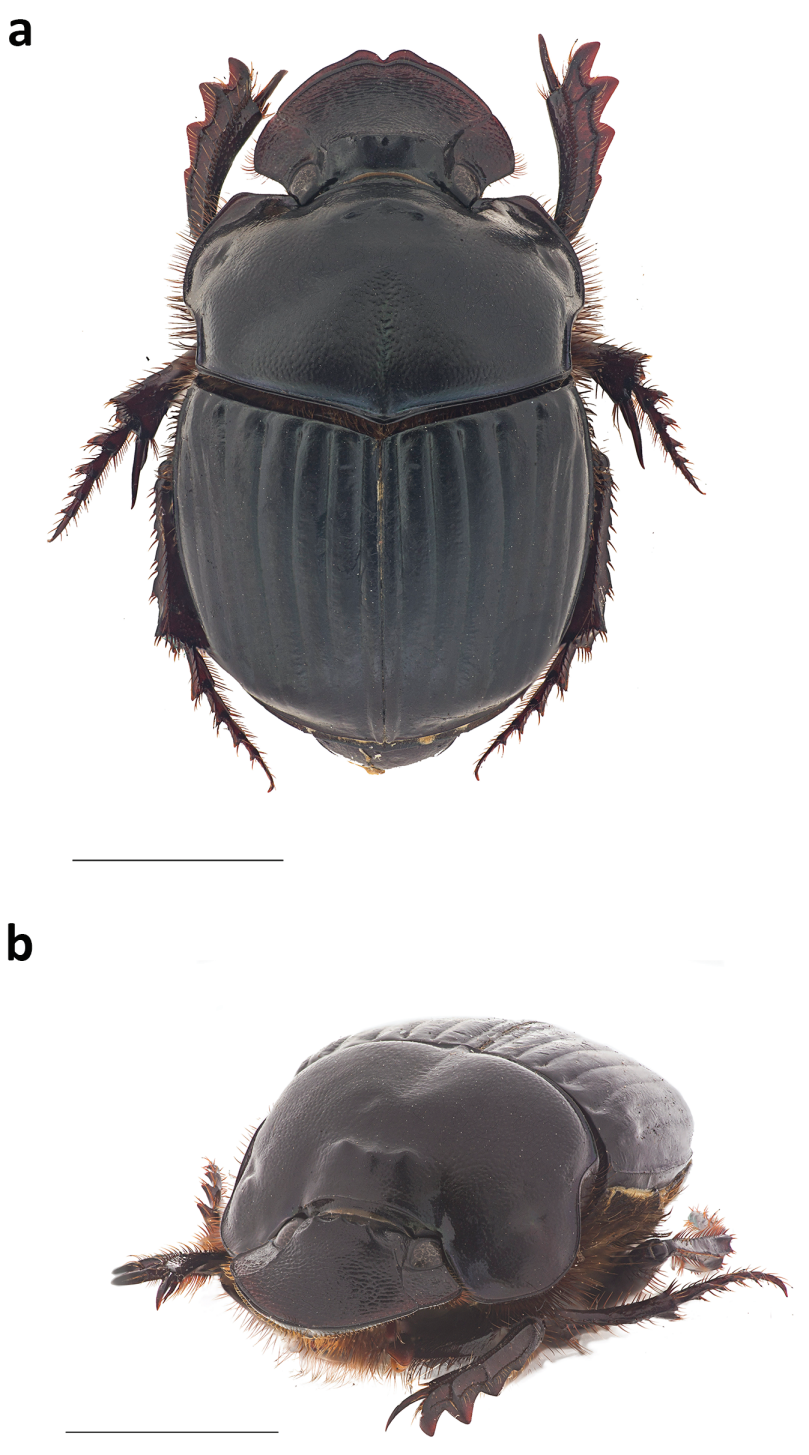
Female habitus of **a** dorsal and **b** lateral view of *Phanaeus dionysius* sp. n. Scale bar= 5mm.

**Figure 5.**
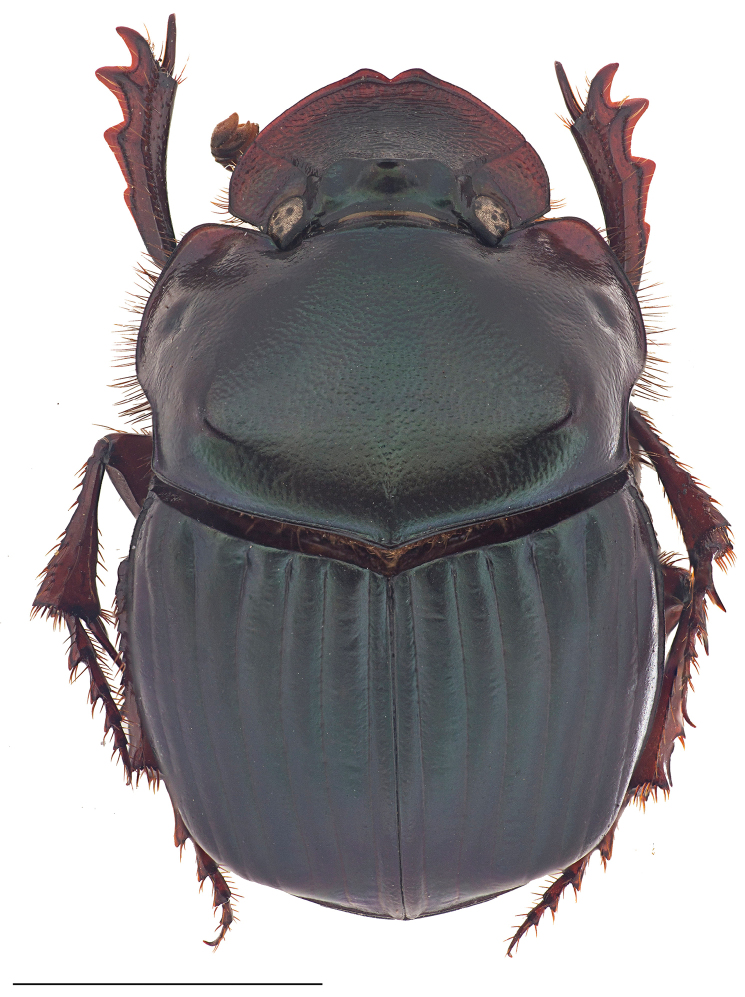
Dorsal habitus of a minor male of *Phanaeus dionysius* sp.n. Scale bar= 5mm.

**Figure 6.**
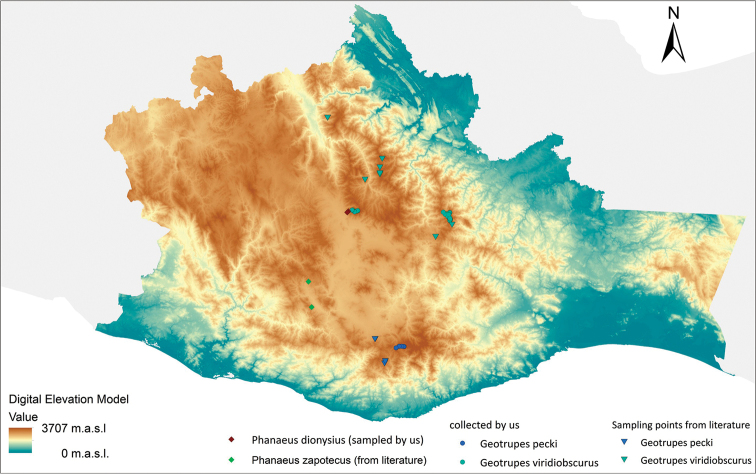
Map of the known distribution of *Geotrupes pecki, G. viridiobscurus*, *Phanaeus dionysius* sp. n., and *Ph. zapotecus*. Orography of Oaxaca is shown, based on the Digital Elevation Model downloaded from INEGI (2017, http://www.inegi.org.mx). Grey area shows the limits of Mexico with the Gulf of Mexico in the North and the Pacific Ocean in the South.

Figures 1 , 2 , 3 , 4 , 5 , 6

#### Type material.

Holotype male, pinned, with genitalia in a separate microvial. Original label: “México. La Mesita San Pablo Etla. Oaxaca.

23-VI-17, coprotrampa, 17°9'54"N, 96°44'18"W, bosque de Encino, 1976 m, Arriaga A. and Arenas A. Col.” “HOLOTYPE/Phanaeus dionysius Kohlmann, Arriaga-Jiménez, Rös [red printed label]”. Allotype female: “Mexico. La Mesita San Pablo Etla. Oaxaca. 23-VI-17, coprotrampa, 17°9'54"N, 96°44'19"W, bosque de Encino, 1976 m, Arriaga A. and Arenas A. Col.”

#### Other material.

(5 males, 5 females). Paratypes: “Mexico. Reserva Comunitaria San Pablo Etla. Oaxaca. 27-IV-17, coprotrampa, 17°9'53"N, 96°44'20"W, bosque de Encino, 1974 m, Arriaga A. and Arenas A. Col.” (1 males, 2 females) (CMN, CEMT, CPFA); “Mexico. La Mesita San Pablo Etla. Oaxaca. 23-VI-17, coprotrampa, 17°9'54"N, 96°44'18"W, bosque de Encino, 1976 m, Arriaga A. and Arenas A. Col. (2 males, 2 females) (CMN, CEMT, IEXA, CPFA); 14-VII-17, 17°9'54"N, 96°44'54"W, 1954 m, (1 male) (IEXA); 27-IV-17, 17°10'16"N, 96°43'50"W, 2219 (1 male) (JB). 23-VI-17, 17°09'54"N, 96°44'19"W, 1976 m, (1 female) (JB).

#### Type locality.

La Mesita San Pablo Etla (17°9'54"N, 96°44'19"W, 1976 m), Oaxaca, Mexico.

#### Type deposition.

Colección Entomológica IEXA, Instituto de Ecología, Xalapa, Mexico.

#### Diagnosis.

Distinctly granulate male pronotal disk; sagittal furrow present on the female pronotum; unmodified sutural margin of the elytra; pygidium longer than wide. Its basal border forming a small indentation medially, usually all-black color.

#### Description.

***Holotype.*** Major male (Fig. 1a–b). Length: 16.5 mm. Humeral width: 10. 9 mm. Body appearing dull shiny black with a faint blue luster to the unaided eye. Magnification reveals faint greenish cast along the ocular, pronotal, and elytral borders, on the abdominal surface and underside of femora rugose. Clypeus with two conspicuous median teeth; surface and frons bearing long, slender horn strongly curved over the pronotum. Pronotum with large, flat triangular disk (Fig. 1a), with a well-developed, small callosity on each side near anterior margin and with postero-lateral angles projecting caudally; lateral portions faintly asperate, with distinct punctures present only behind lateral fossae (×20); flat triangular surface disk densely, evenly, and coarsely granulate; granules extending onto posterolateral angles and becoming eroded near and along disk borders. Pronotum with obsolete basal fossae; anterolateral angles subquadrate, distinctly upturned and surface behind angles concave; pronotal midline present, faintly developed, more evident on anterior half; weak punctures along posterior pronotal margin; pronotal surface shagreen. Elytral striae fine, with small but well-defined punctures separated at regular intervals; intervals broad and faintly convex, evenly and faintly shiny, covered with minute punctures (×20); surface shagreen. Pygidium black with shagreen surface and obsolete punctures, glabrous; pygidium wider than long (Fig. 2a); basal pygidial margin forming a small triangular tooth medially (Fig. 2b); pygidial margin with a green cast. Protibia quadridentate. Lamella copulatrix as in Figure 3a; aedeagus similar to the *Ph. endymion* species group (Fig. 3b, d).

***Female.* Allotype** (Fig. 4). Length: 16.3 mm. Humeral width: 10 mm. Body faintly shining black. Head with low, narrow trituberculate carina. Pronotum with a faint green lustre, evenly and densely covered with punctures, punctures becoming fainter on middle of disk; surface shagreen; with raised anteromedian trituberculate tumosity near anterior margin, tubercles equal in size and set in a more-or-less straight, transverse line; disk with distinct mid-longitudinal furrow, extending forward from posterior margin to about middle of disk, furrow more strongly sculptured than adjacent surface of disk. Pygidium with faint to distinct fine, sparse punctures.

#### Variations.

Length: 12.6–18.7 mm. Humeral width: 7.9–11.1 mm. Pronotal disk of males may vary from black without reflections to having a green or red lustre. Minor male (Fig. 5): Similar to major male, except the cephalic horn is smaller and the posterolateral angles of the pronotum are reduced.

#### Etymology.

Due to the fact that this species has been collected in association with *Ph. damocles* Harold, this new species (a noun in the nominative singular) is named after Dionysius II of Syracuse, one of the main characters alluded to in the moral anecdote of the “Sword of Damocles”.

#### Distribution and ecology.

**Figure 7.**
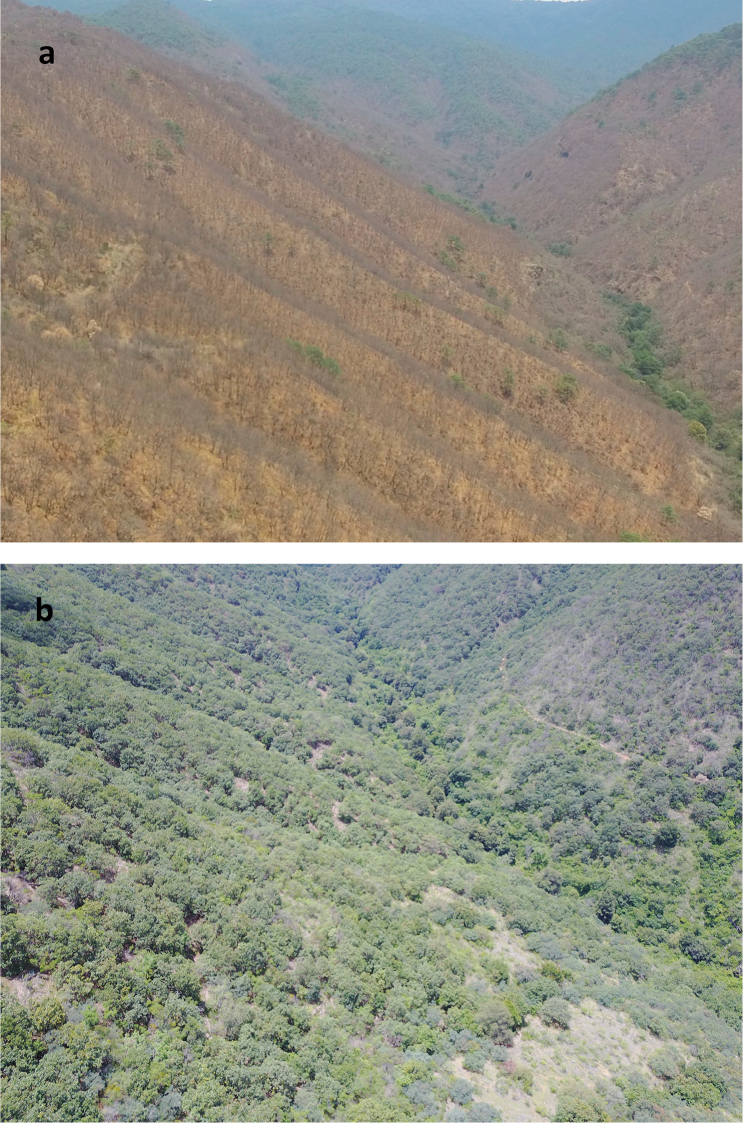
Drone photographs of the dry oak forest where *Ph. dionysius* sp. n. was collected; **a** April 2017 and **b** August 2017.

So far, this species is only known from San Pablo Etla in the Sierra Norte (Sierra de Ixtlán) in Oaxaca (Fig. 6), along the internal dry slope facing the Oaxaca Valley. It has been collected from altitudes of 1950 m to 2250 m. The dry deciduous oak forest where *Phanaeus dionysius* sp. n. was found is characterised by trees between five and ten meters tall. Abundant oak species are *Quercus laeta* Liebm. and *Q. laurina* Humb. and Bonpl., predominant species of this ecosystem, ranging from 1800 m to 2400 m altitude (Fig. 7). Other species dominating this forest in the sampling site are *Q. glaucoides* Mart. and Gal., *Q. liebmannii* Oersted., *Q. rugosa* Née, and *Q. castanea* Née, also found at higher or lower altitudes (J. Williams, CIIDIR-Oaxaca, *pers. comm*., Valencia-Ávalos and Nixon 2004). This dry deciduous oak forest shows a strong seasonality, when most trees lose their leaves for around four to five months between December and May. This new species is found next to the Oaxaca Metropolitan Area in a voluntary protected area, the San Pablo Etla Community Reserve “La Mesita”.

*Phanaeus dionysius* sp. n. has been collected simultaneously in dung-baited traps with *Canthidium quercetorum* Kohlmann, Arriaga-Jiménez and Rös, *Canthon humectus* (Say), *Copris klugi* Harold, *Deltochilum mexicanum* Burmeister, *Dichotomius colonicus* (Say), Onthophagus near anthracinus Harold, *O. aureofuscus* Bates, *O. chevrolati retusus* Harold, *O. mexicanus* Bates, *O. zapotecus* Zunino and Halffter and *Phanaeus damocles* Harold, in the oak forest. Although its closest relative, *Ph. zapotecus* Edmonds, seems to be a strictly mycetophagous species, *Ph. dionysius* has only been collected in dung, despite the presence of fungi-baited traps put in the forest. Interestingly, no big fleshy fungi (toadstools) were observed in this type of forest, only small “clavitos” (*Lyophyllum*), which could probably explain why this species does not exploit fungi.

#### Taxonomic relationships.

*Phanaeus dionysius* sp. n. belongs to the *Ph. endymion* species group and due to its close taxonomic similarity discussed below is postulated to be the sister species of *Ph. zapotecus* Edmonds, 2006. *Phanaeus dionysius* will key out to *Ph. zapotecus* in the key of Moctezuma et al. (2017) and can be separated from it because it has long and slender pronotal posterolateral angles (Fig. 1a) whereas *Ph. zapotecus* has short and rounded posterolateral angles (Fig. 1c). The basal border of the pygidium in *Ph. dionysius* forms a small indentation at its middle (Fig. 2b), whereas it runs completely straight in *Ph. zapotecus* (Fig. 2c). Additionally, the apex of the parameres of *Ph. dionysius* sp. n. is more projected (Fig. 3b, d), than that from *Ph. zapotecus* (Fig. 3c, e). Moreover, the middle sinuation of the parameres in lateral view is much more pronounced in *Ph. dionysius* sp. n. (Fig. 3b) than in *Ph. zapotecus* (Fig. 3c).

#### Chorological affinities.

The known distribution of *Ph. dionysius* sp. n. is relatively near to its closest relative, *Ph. zapotecus*, 90 km distance in a straight line, which is distributed in dry pine-oak and pine-oak-juniper forests on the internal slope of the Sierra Sur (Sierra de Tlaxiaco), going from 1850 m to 2150 m altitude. Interestingly, attempts at trying to collect *Ph. zapotecus* in the environs of San José del Pacífico in the Sierra Sur (Sierra de Miahuatlán) with fungi-baited traps did not produce any results.

### Species revalidation

#### Phanaeus (Notiophanaeus) malyi

Taxon classificationAnimaliaColeopteraScarabaeidae

Arnaud, 2002

Fig. 8a–b

Phanaeus pyrois malyi : Arnaud (2002: 96–97).
Phanaeus malyi Arnaud, Solís & Kohlmann (2012: 10).
Phanaeus pyrois Bates, Edmonds & Zídek (2012: 1, 3, 13, 52–53, 57)

##### Remarks.

**Figure 8.**
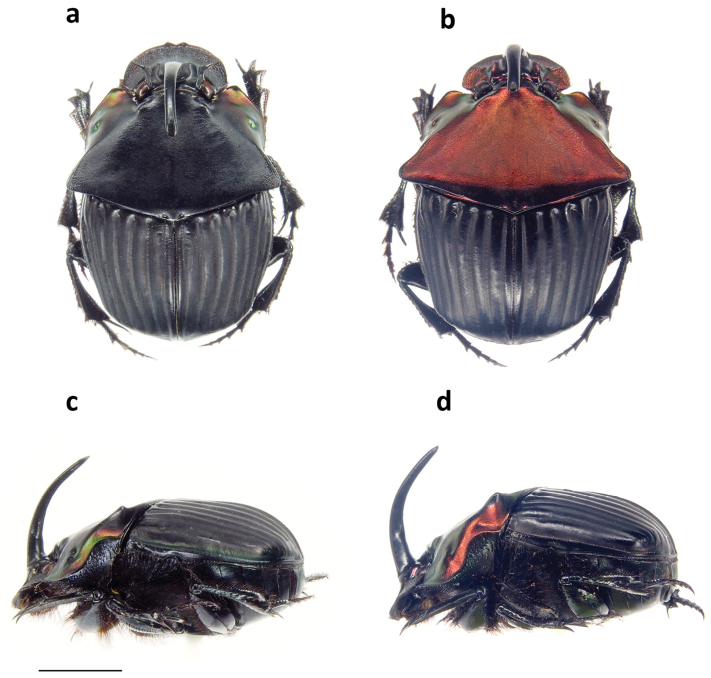
Major male habitus in **a** dorsal and **b** lateral view of *Phanaeus malyi*, and in **c** dorsal and **d** lateral view of *Ph. pyrois.* Scale bar= 5mm.

Solís and Kohlmann (2012) elevated *Phanaeus pyrois malyi* Arnaud to species status based on the results of mitochondrial DNA studies of Costa Rican specimens that clearly separated Caribbean from Pacific populations at a genetic distance concordant with species level (average Kimura-2-parameter [K2P] = 3.8 %, Solís and Kohlmann 2012). Moreover, *Ph. malyi* has short and rounded postero-lateral pronotal angles (Fig. 8a–b), whereas they are long and slender in *Ph. pyrois* (Fig. 8c–d). Subsequently, Edmonds and Zídek (2012) synonymised *Ph. pyrois malyi* with *Ph. pyrois*, pending a comparative analysis of black varieties of Panamanian and South American populations. *Phanaeus malyi* was compared with material from J. Blackaller’s private collection and photos sent by François Génier from the Canadian Museum of Nature (Suppl. material 1: Figure S1) of black specimens of *Ph. pyrois* from Cerro Campana, Province of Panama, Panama (formerly treated as *Ph. pyrois olsoufieffi* Balthasar) and Playa de Oro, Esmeralda, Ecuador (formerly treated as *Ph. pyrois funereus* Balthasar). *Ph. malyi* and *Ph. p. olsoufieffi* are different taxa. While *Ph. malyi* has short and rounded posterolateral angles of the pronotum, similar to *Ph. endymion*, *Ph. p. olsoufieffi* has the typical *pyrois* long and slender posterolateral angles of the pronotum. *Ph. p. olsoufieffi* also exists in Costa Rica, distributed along the foot of the mountains and not in the lowlands like the typical metallic-red *pyrois*. A more detailed analysis would be required in order to determine if it should again be resurrected into subspecific status. In relation to *Ph. p. funereus*, these specimens have an intermediate form of the postero-lateral angles of the pronotum between *malyi* and *olsoufieffi*. Moreover *funereus* is distinctly dull black whereas *malyi* is a shiny black. A more detailed analysis is required for resurrecting *funereus*, but it is a different taxon from *malyi*.

We therefore revalidate *Ph. malyi* to full species status based on the previously mentioned mitochondrial DNA analysis (Solís and Kohlmann 2012); the characteristic ecology and distribution of this species in tropical rainforest along the southern Pacific coast of Costa Rica, probably extending to the northern Panamanian Pacific coast (Solís and Kohlmann 2012); and the different shapes of the pronota (Suppl. material 1: Figure S1).

*Phanaeus malyi* follows a characteristic distribution pattern described by Kohlmann and Wilkinson (2007) and by Solís and Kohlmann (2012), where many species living in the Costa Rican southern Pacific tropical forest are sister species of taxa found along the tropical forest of the Caribbean coast. This vicariant speciation pattern (apparently mediated by the emergence of the Talamanca Cordillera that has divided a formerly continuous tropical forest stretching from the Caribbean to the Pacific) has been observed to occur in species of dung beetles, fishes, amphibians, reptiles, and birds (Kohlmann and Wilkinson 2007).

### New localities of the *Phanaeus endymion* group

#### Phanaeus (Notiophanaeus) bravoensis

Taxon classificationAnimaliaColeopteraScarabaeidae

Moctezuma, Sánchez-Huerta & Halffter, 2017

Phanaeus bravoensis : Moctezuma et al. (2017: 115, 118–122).

##### Remarks.

Recently this species has been described as found in Guerrero. Here we complement its poorly known distribution with new localities from the Blackaller private collection:

Guerrero (38 specimens): S. del Alquitrán, 1470 m 22-VI-1990 Luz L. Delgado, J. Blackaller col.; Palo Blanco, 20-VI-1990 Alt. 1400 m Bosque de pino-encino; Bosque Mesófilo, Alt. 1,710 mts. Coprotrampa exc. humano, 28 jun-24 jul/2055 EPE 5 mts., 17°22'N, 99°30'W L. Muñoz and J. Blackaller col.; Pino-Encino, alt. 1100-1380 m, En hongos (dentro de galería), 31/Jul/2004, 17°21'N, 99°30'W; 1100–1250 mts., 11/Jul/2004, 17°21'N, 99°29'W. Mochitlán, San Roque (8 km al este de Acahuizotla) Bosque Tropical y Bosque de Encino, Alt. 1130 m, CD en hongos, Ago-sept. 2013, 17°22'N, 99°25'W EPE 5 mts. Blackaller col.; (8 km al noreste de Ximilcotitlán), Bosque de Pino y de Encino, Alt. 2140 m, 7-VIII-2015 CD en hongos, 17°23'N, 99°23'W; 2 km al este de Ximilcotitlán, Alt. 1680 m, 07-08-VI-2009, en hongos, 17°22'N, 99°25'W; 5 km al este de Acahuizotla (camino a San Roque), Bosque de Encino y ET, Julio-2007, Alt 1010 msnm Trampa de luz CG 17°21'N, 99°26'W.

Moctezuma et al. (2017) report this species seemingly copro-necrophagous. It is reported here to be also mycetophagous and living not only in pine-oak forest, but also in the cloud forest.

#### Phanaeus (Notiophanaeus) halffterorum

Taxon classificationAnimaliaColeopteraScarabaeidae

Edmonds, 1979

Phanaeus halffterorum : Edmonds (1979: 99; *partim*), Halffter and Edmonds (1982: 88–89), Anduaga and Halffter (1991: 157), Delgado-Castillo et al. (1993: 125), Edmonds (1994: 39–43, 101), Anduaga (2000: 125, 130), Arnaud (2002: 95– 96), Edmonds (2003: 61, 65), Edmonds (2006: 31, 34, 36), Ceballos et al. (2009: 397), Edmonds and Zídek (2012: 3, 5, 12, 52, 54), Deloya et al. (2014: 77), Moctezuma and Halffter (2017: 52, 54–55), Moctezuma et al. (2017: 115), Lizardo et al. (in press).

##### Remarks.

This study identifies new localities of this species present in the Blackaller private collection, which seems to have a rather restricted distribution in the State of Mexico.

Estado de México (8 specimens): Sierra de Nanchititla, 6 km al Este de Nanchititla, Bosque de Pino-Encino, CD en Hongos, 1840 m, 15-VIII-2015, 18°52'N, 100°24'W, J.Blackaller y L. Zacarías cols.; 06-VIII-2011, Trampa de Luz, Blackaller y Robacker cols.; 21-VIII-2011, J. Blackaller col.

Comparing this species with its closest relative, *Ph. bravoensis*, one can add as a further difference between the two species the fact that *Ph. halffterorum* has its pygidium covered with coarse punctures while *Ph. bravoensis* has faintly impressed small punctures. Although it is not specified in the original description of this species (Edmonds 1979), specimens were collected directly by hand feeding on fungi pertaining to the genus *Boletus*.

#### Phanaeus (Notiophanaeus) huichol

Taxon classificationAnimaliaColeopteraScarabaeidae

Moctezuma, Sánchez-Huerta & Halffter, 2017

Phanaeus huichol : Moctezuma et al. (2017: 123-129).

##### Remarks.

Recently this species has been described as from Jalisco and Nayarit. We register here the northernmost and first locality of this species from the state of Sinaloa using material from the Blackaller private collection:

MEXICO (1 male, 1 female). Sinaloa. La Venada. 4 km al Noroeste de Microondas. Loberas. Bosque de pino-encino. 1780 msnm en excremento. 16-18/VIII/2007 EPE 5 metros CG 23°30'N, 105°52'W Blackaller y Folschveiller Cols.

### Key to the *Phanaeus endymion* species group (modified from Edmonds and Zídek 2012 and Moctezuma et al. 2017)

**Table:** 

1	Sutural margin of each elytron upturned to form a sharp ridge, which is progressively more elevated posteriorly and prolonged into a small, sharp tooth at apical angle; elytral margin slightly excised adjacent to this tooth	**2**
–	Sutural margin of elytra simple. Color and distribution variable	**3**
2	Major male with a tooth in the middle of anterior pronotal margin, pronotal triangle sides’ straight, pygidium covered with coarse punctures. State of Mexico	***halffterorum* Edmonds**
–	Major male lacks a tooth in the middle of anterior pronotal margin, pronotal triangle sides’ curved, pygidium covered with faintly impressed small punctures. Central Guerrero	***bravoensis* Moctezuma, Sánchez-Huerta & Halffter**
3	Triangular pronotal disk of male evenly and densely but finely granulated (×10), granules in most specimens larger and becoming squamose along lateral margins of disk and extending onto posterolateral angles (when distinctly developed); sides of pronotum roughened (×10), lacking distinct punctures except behind lateral fossae. Female pronotum minutely roughened, evenly, distinctly punctate (×10), punctures becoming smaller dorsally but not disappearing altogether; disk impressed medially as a distinct furrow visible to unaided eye, extending forward from posterior margin to near middle of disk. Oaxaca	**4**
–	Pronotal disk of male either lacking distinct granulation, or, if granules present, these are minute and restricted along lateral margins of disk; sides of pronotum smooth, minutely punctate. Female pronotum smooth, punctures (×50) fine and usually restricted to sides; median furrow lacking or at most indicated by a fine, scarcely visible line	**5**
4	Major males with long and slender posterolateral angles of pronotum (Fig. 1a); pygidium has its basal border forming a small indentation at its middle (Fig. 2b); aedeagus and lamella copulatrix as in Fig. 3a, b, and d. Sierra Norte (Sierra de Ixtlán)	***dionysius* sp. n.**
–	Major males with short and rounded posterolateral angles of pronotum (Fig. 1c); basal border of the pygidium does not form a small indentation at its base (Fig. 2c); aedeagus as in Figs 3c and d. Sierra Sur (Sierra de Tlaxiaco)	***zapotecus* Edmonds**
5	Elytral interstriae distinctly flattened and uniformly dull (more convex and shiny in some Central American populations); striae not strongly impressed basally, anterior ends in most specimens bearing deep punctures rather than large fossae. Male: Pronotal disk dull, velvety smooth medially, finely asperate, brighter laterally. Female: Pronotum evenly convex, lacking anteromedial concavity even in largest specimens, bearing three round, smooth tubercles in transverse line near anterior margin. Head and pronotum highly shiny metallic red or green to nearly completely dull black with metallic red restricted to ridges and isolated areas on anterior part of pronotum; elytra dull to lightly shiny black; pygidium usually metallic red medially, green peripherally, in some completely red or green. Southern Nicaragua through Central America into western Colombia and Ecuador	**6**
–	Elytral interstriae evenly convex and glossy midlongitudinally; striae impressed basally as distinct fossae. Male: Pronotal disk velvety smooth medially, finely asperate laterally and sometimes also medially. Female: Pronotum with anteromedial concavity bounded anteriorly by a raised U- or V-shaped ridge	**8**
6	Major males with short and rounded posterolateral angles of pronotum (Fig. 8a). Body black. Rain forest Pacific slope of Costa Rica and Panama	***malyi* Arnaud**
–	Major males with long and slender posterolateral angles of pronotum (Fig. 8c). Body black or with head and pronotum metallic red, green or blue and black or dark green elytra. Nicaraguan Caribbean slope to Ecuador	**7**
7	Head and pronotum green or blue with dark green elytra; male pronotum with sparse and blunt granulation on disc; female pronotal trituberculate ridge forming a triangle. Ecuador	***arletteae* Arnaud**
–	Body black or with head and pronotum metallic red or green and black elytra; male pronotum with fine reticulation on disc; female pronotal trituberculate ridge forming a transverse line. Nicaragua to Ecuador	***pyrois* Bates**
8	Dorsum dark blue or shiny green; in few specimens shiny green with strong yellow reflections. Anterior margin of pronotum projected forwards. Relatively rounded posterolateral angles of pronotum. Southwestern Mexico to Honduras	***endymion* Harold**
–	Dorsum metallic green. Anterior margin of pronotum projected upwards. Acute posterolateral angles of pronotum	**9**
9	Anterior metasternal angle obtuse in lateral view. Lateral metasternal angles well defined and slightly curved. Few specimens olive green with golden/reddish reflections. Eastern Oaxaca and western Chiapas	***zoque* Moctezuma & Halffter**
–	Anterior metasternal angle almost right angled but with rounded apex in lateral view. Lateral metasternal angles evanescent. Jalisco and Nayarit	***huichol* Moctezuma, Sánchez-Huerta & Halffter**

### Distribution and new localities of *Geotrupes* in Oaxaca, Mexico

#### Geotrupes (Onthotrupes) pecki

Taxon classificationAnimaliaColeopteraScarabaeidae

Howden, 1974

Figs 6 , 9a–b , 10b

Geotrupes pecki : Howden (1974: 572, Plate II), Trotta-Moreu et al. (2008: 43, 47, 50, 51), Trotta-Moreu and Lobo (2010: 46).

##### Remarks.

This species has been recorded only once in the literature, more than 40 years ago, when Howden described it in 1974 in the Sierra Sur (Sierra de Miahuatlán). Using the only two known distribution records reported by Howden (1974), Trotta-Moreu et al. (2008) performed an analysis of the known and potential distribution of *G. pecki* in the state of Oaxaca using the MaxEnt prediction model (Phillips et al. 2006). For *G. pecki*, Trotta-Moreu et al. (2008) estimated a potential distribution area of 5254 km^2^ and mean values for altitude (1862 m), rainfall (1524 ±287 mm), and temperature (18.5 ±5 °C). With the present collecting localities, we can recalculate and correct the Trotta-Moreu et al. (2008) too low mean value for altitude for this species.

Below, an updated key for the genus *Geotrupes* in Oaxaca is presented. *G. truncaticornis* Howden is not included in the key; because it is known only from Guerrero. Trotta-Moreu et al. (2008) erroneously reported it from Oaxaca (Kohlmann *in press*).

**Material** (6 males, 8 females). Holotype: México.10 km E Sn Sebastián Río Hondo, Miahuatlán, Oaxaca. 31-VIII-17, coprotrampa, 16°11'56"N, 96°21'54"W, bosque pino/pingüica, 2930 m, Arriaga J. A. Col. Allotype: México. 4 km NO Sto. Domingo Ozolotepec, Miahuatlán, Oaxaca. 31-VIII-17, coprotrampa, 16°11'57"N, 96°21"W, bosque pino/pingüica, 2920 m, Arriaga J. A. Col. Paratypes: México. 4 km NW Sto. Domingo Ozolotepec, Miahuatlán, Oaxaca. 31-VIII-17, coprotrampa, 16°11'56"N, 96°21'56"W, bosque pino/pingüica, 2930 m, Arriaga J. A. Col. (1 male, 1 female), 16°11'57"N, 96°21"W, 2920 m, (one male, three females); 3 km NW Sto. Domingo Ozolotepec, 16°11'53"N, 96°20"W, 2740 m, (2 males); 8 km E San Sebastián Río Hondo, 30-VIII-17, C. D. exc. Vaca, 16°11'15"N, 96°23'15"W, 2900 m, (1 male, 3 females).

#### Geotrupes (Onthotrupes) viridiobscurus

Taxon classificationAnimaliaColeopteraScarabaeidae

Jekel, 1865

Figs 6 , 9c , 10a

Geotrupes viridiobscurus : Jekel (1865: 599), Bates (1887: 113), Howden (1964: 50, 52-53, 87), Howden (2003: 97), Martínez and Suárez (2006: 778), Trotta-Moreu et al. (2008: 43, 47, 50, 51), Trotta-Moreu & Lobo (2010: 46), Alvarado et al. (2013: Supplemental material).
Geotrupes saundersi : Jekel (1865: 568), Bates (1887: 113).
Geotrupes felschei : Nonfried (1894: 114).

##### Material.

Mexico. Oaxaca. Zempoaltéptl, 27-V-17, coprotrampa, 17°7'5"N, 96°0'W, matorral/pastizal, 3040 m Arriaga A. and Arenas A. Col.; 30-V-17, bosque de pino/aile 3190m; 17°9'7"N, 96°1'29"W, bosque de encino 2850 m. Reserva Comunitaria San Pablo, Etla, 20-IX-16 coprotrampa, 17°16'70"N, 96°68'55"W, bosque de pino 2980 m; 17°16'72"N, 96°68'50"W, 2980 m; 17°17'14"N, 96°67'26"W, 3070 m; 17°17'70"N, 96°67'27"W, 3110 m; 17°17'12"N, 96°67'20"W, bosque de pino 3100; 23-IX-16, 17°17'84"N, 96°70'38"W, bosque pino/encino 2700 m. Yucuiji, San Esteban Atatlahuca, 18-08-17, exc. caballo, 17°07'32"N, 97°40'44"W, bosque de pino 3150 m. La Chinantla, 17°35'48"N, 96°28'20"W, 2200 m; 17°35'12"N, 96°29'21"W, 2400 m; 17°34'48"N, 96°29'43"W, 2600 m. El Llano de las Flores, 17°45'N, 96°50'W, 2800 m.

##### Historical.

Oaxaca. Duraznal, 17°1'55"N, 96°10'12"W, 1820 m.

##### Notes.

So far, this species has only been collected in Oaxaca and seems to be restricted to the Sierra Norte (Sierra de Ixtlán, Sierra Mazateca, and Nudo del Zempoaltépetl) in the northern part of the state. Howden (1964) cites only two historical localities for the state taken from Bates’ (1886-1890) work, Duraznal, Oaxaca, and (La) Parada. The latter we have been unable to locate. Martínez and Suárez (2006) subsequently cited the locality of “El Llano de las Flores” for this species. Trotta-Moreu et al. (2008) published a study on the known and potential distribution of Geotrupidae in Mexico. Using the GEOMEX database, they predicted a potential distribution based on six localities (which are not cited in their text) and calculate mean values of temperature (15.4 ±3 °C), precipitation (1357 ±936 mm) and altitude (1793 m), as well as a potential distribution area (3352 km^2^) for this species in the state of Oaxaca. They ambiguously cite that this species is distributed in the Sierra Madre del Sur, not specifying that actually it is distributed in the Sierra Norte. A recent book on the biodiversity of the Sierra Madre del Sur by Luna-Vega et al. (2016) implies that all mountains in Oaxaca belong to the same biogeographic region called Sierra Madre del Sur. This is not a correct biogeographic regionalisation, as the results of this present paper and another (Kohlmann in press) show great differences in the dung beetle fauna of the Sierra Norte and the Sierra Sur. The Sierra Norte shows considerable influence from elements stemming from the Sierra Madre Oriental and the Trans Mexican Volcanic Belt (Kohlmann in press). Finally, Alvarado et al. (2013) cite three georeferenced localities at La Chinantla. With the present collecting localities, we can recalculate and correct the Trotta-Moreu et al. (2008) too low mean value for altitude for this species.

##### Taxonomic relationships.

**Figure 9.**
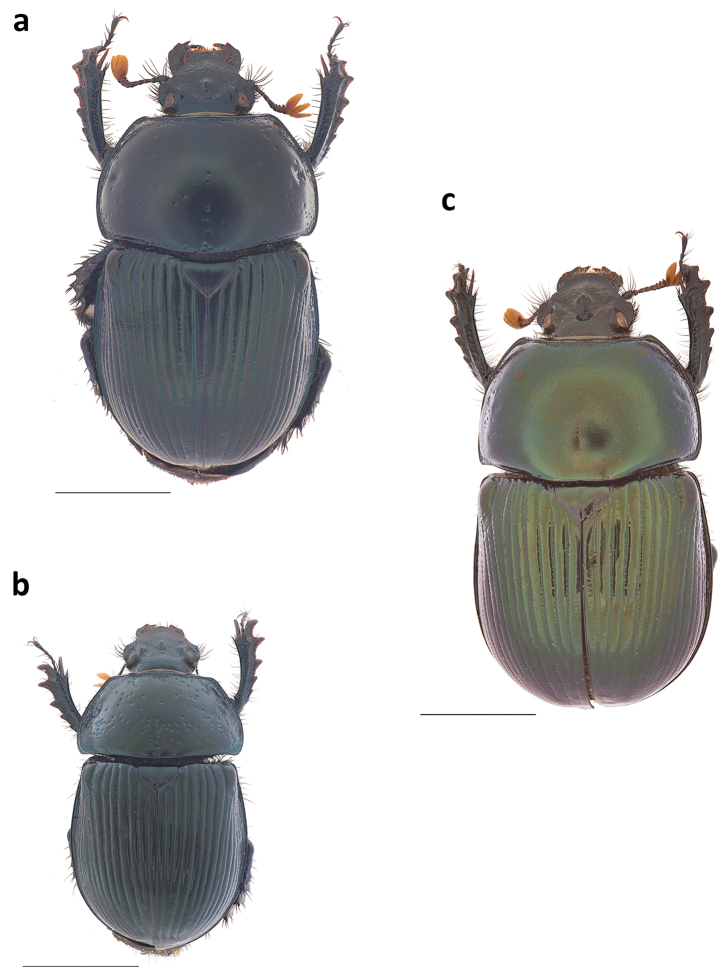
Dorsal habitus of **a** a male of *G. pecki* **b** of a female of *G. pecki*, and **c** of a male of *G. viridiobscurus*. Scale bar= 5mm.

**Figure 10.**
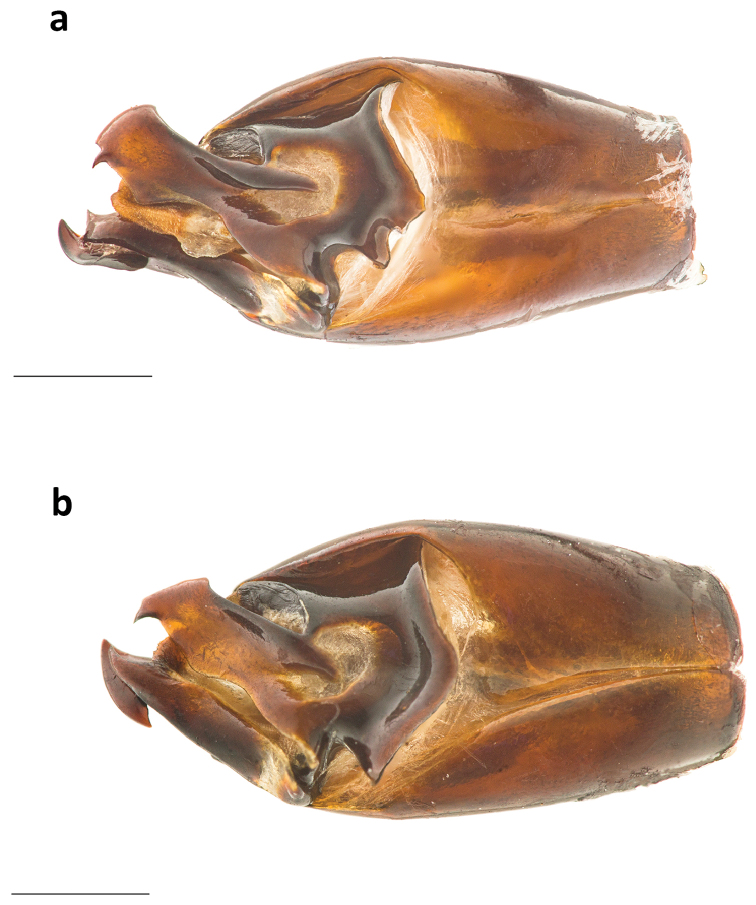
Aedeagi of **a** *G. viridiobscurus* and **b** *G. pecki*. Scale bar= 1mm.

*Geotrupes pecki* and *G. viridiobscurus* have very similar aedeagi with only small differences on the parameres, as can be observed from the photographs of their genitalia (Fig. 10). They also present small differences in the elytral striae, being more or less punctate or crenulated as well as having a more or less punctate pronotum, over the whole surface (*pecki*) or concentrated at the pronotal sides (*viridiobscurus*). Howden (1974) has indicated that *G. pecki* has a heavily punctate pronotum, but as Fig. 9 clearly shows, this is not always the case and the pronotum can resemble the one of *G. viridiobscurus*. Due to the significant taxonomic similarity discussed above it is here postulated that this species pair are sister species.

##### Chorological affinities.

The known distribution of *G. viridiobscurus* in the Sierra Norte is relatively adjacent to its closest taxonomic relative, *G. pecki*, in the Sierra Sur, only 125 km away in a straight line (Fig. 6). Both species are distributed in pine and pine-oak forests; *G. viridiobscurus* also has been collected in oak and pine/alder forest. *G. pecki* has been collected from 2400 m to 3000 m altitude whereas *G. viridiobscurus* has been collected from 2000 m to 3200 m altitude (the Duraznal historical record with 1800 m altitude does not represent a precise locality). The Trotta-Moreu et al. (2008) mean altitude calculations of 1800 m for both species may be incorrect, and therefore it is probable that their calculations of the other climatic variables (temperature and precipitation), and distribution predictions, also would be erroneous.

### Key to the *Geotrupes* of Oaxaca (modified from Howden 1964, 1974)

**Table:** 

1	Antennal club grayish-black; each mandible with a very pronounced rounded lobe. Sierra Sur (Sierra de Miahuatlán)	***lobatus* Howden**
–	Antennal club yellowish or reddish brown to brown; mandibles lacking a very pronounced rounded lobe	**2**
2	Dorsally shining, often green or blue, elytral intervals convex	**3**
–	Dorsally dull black; elytral intervals flattened centrally; large species with males having the fore femora ventrally excavated near the coxae; mountains in central Mexico and Sierra Norte (Sierra de Ixtlán)	***sallei* Jekel**
3	Posterior pronotal margin fine but distinct, except in front of scutellum. Sierra Norte (Sierra de Ixtlán)	***nebularum* Howden**
–	Posterior pronotal margin indistinct or lacking in front of third to seventh elytral striae	**4**
4	Elytral striae finely punctate or crenulated except near suture; elytra normally greenish-black, sometimes with reddish tint; pronotum punctate laterally (Fig. 9a); aedeagus like Fig. 10a. Sierra Norte (Sierra de Ixtlán, Sierra Mazateca, and Nudo del Zempoaltépetl)	***viridiobscurus* Jekel**
–	Elytral striae virtually impunctate, at most vaguely crenulate; elytra black with faint tinge of green on elytron; pronotum generally heavily and grossly punctate (Fig. 9b); aedeagus like Fig. 10b. Sierra Sur (Sierra de Miahuatlán)	***pecki* Howden**

## Discussion

Edmonds (1994) comments that the *endymion* group of the subgenus Notiophanaeus represents a recent migration into Middle America and spread in response to the northward expansion of Neotropical forests during the Pleistocene. At the time of his first study (Edmonds 1994) only two species (*endymion* Harold and *halffterorum* Edmonds) of this group were known to exist in Mexico. At present we know that there are actually seven species (*bravoensis, dionysius* sp. n., *endymion, halffterorum, huichol, zapotecus, zoque*) belonging to the *endymion* group distributed in Mexico (out of the ten [*arletteae*, *malyi*, *pyrois*] that comprise the whole group), which prompts us to reassess the probable time of entry of this group into Mexico. These species inhabit a plethora of ecosystems, such as dry oak scrub-forest, dry pine-oak and dry pine-oak-juniper forest, cloud forest, and wet pine-oak forest. In the case of Oaxaca, Cevallos-Ferriz and Ramírez (2004) indicate the presence of *Pinus* during the Eocene-Oligocene in the neighbouring state of Puebla and of cloud forest during the Miocene. *Quercus* is registered for the lower Oligocene in the neighbouring state of Puebla, appearing during the Miocene in Oaxaca. Considering the previous facts and that some of the species have become specialised in mycetophagy and the group’s distribution reaches to the state of Sinaloa, this suggests that the *endymion* group spread to Middle America around the Miocene. This would have occurred concomitantly with the estimated spread of the subgenus Phanaeus, as Edmonds (1994) has postulated; and not during the Pleistocene, as Edmonds (1994) had previously suggested for the subgenus Notiophanaeus. However, closely related species pairs, like *halffterorum*-*bravoensis* and *zapotecus*-*dionysius* probably speciated recently, as a possible product of the last glaciation cycles, possibly after the Last Glacial Maximum (LGM) (22 ky to 18 ky cal BP, Caballero et al. 2011), as the small morphological differences between these two species pairs suggest.

Caballero et al. (2011) indicate in a very interesting study that as the LGM temperatures descended to between 6 and 8 °C, the Permanent Snow Line (PSL) descended 1000 m to 3940 m in the Iztaccíhuatl volcano and 3400 m in the Tancítaro and 3650 m in the Cofre de Perote volcanoes. Based on the results, this same study (Caballero et al. 2011) proposes that the alpine meadow/*Pinus* border descended by ~1000 m along with distributional and compositional vegetation changes. At the beginning of the Terminal Glacial (15 ky to12 ky cal BP), all the glaciers retreated slowly and intermittently, then markedly after 14 ky cal BP, creating drier conditions in the internal basins of the centre of Mexico. At present, the upper limit of the *Pinus* forest in the Iztaccíhuatl volcano is located at 4020 m (Caballero et al. 2011). *Pinus* is established in Mexico at broad temperature (15 °C±10 °C) and precipitation (800 ±150 mm) ranks (Velázquez et al. 2000). Caballero et al. (2011) indicate the existence of a precipitation gradient going from both coasts in Mexico to the inland; therefore, glaciers in the inland (Iztaccíhuatl) did not descend as in mountains with a greater maritime influence (Tancítaro and Cofre de Perote), indicating the existence of less humid environments in the inland regions of central Mexico. An intense deglaciation started in the mountains of central Mexico between 15ky and 14 ky cal BP and continued to about 12. 5 ky cal BP (Caballero et al. 2011). Caballero et al. (2011) indicate that during the LGM, the *Pinus* upper limit in the mountains of central Mexico descended from 4020 m to ~3000 m and the upper limit of the *Quercus* forest from 3050 m to ~2150 m, based on a 6°C drop in temperature and a descent of ~1000 m of the PSL.

In relation to this lowering of the glaciers during the LGM, Metcalfe (2006) suggests that in central Mexico glaciers and alpine grasslands expanded and there were extensive forests of pine, oak, spruce, and alder. This forest expansion during the LGM allows us to propose a scenario where the mother species of *Phanaeus dionysius-zapotecus* and of *Geotrupes pecki-viridiobscurus* occupied a continuous forest condition in the central highlands of Oaxaca, framed by the borders of the Sierra Norte to the North and the Sierra Sur to the South (Fig. 6). Both species pairs are related very closely taxonomically, as suggested in the above-mentioned taxonomic treatment, and also are geographical neighbours. A similar scenario could also be proposed for the mother species of *Phanaeus bravoensis-halffterorum* (another very closely related species pair); in this case framed by the Mexican Transvolcanic Belt to the North and the Sierra Madre del Sur to the South in the State of Mexico-Guerrero area. The continuity of these forests could have been broken up after the LGM, when glacier lower limits started receding to their present higher elevations following the concomitant increase in temperatures and temperate Nearctic forest movement into higher altitudes. This situation possibly could have initiated a vicariant speciation process as suggested by the small taxonomic differences reported for these species pairs in the present study. This mechanism would be following the sky-islands dynamic (Knowles 2000) where species persist in high-elevation refugia during the interglacials, having a higher probability of diverging, enhanced by reduced gene flow and contrariwise presenting genetic admixture at lower elevations during glacial periods. This proposal is a novel approach for studying and understanding speciation patterns for closely related mountain species in southern Mexico. This study is intended as a taxonomic reference for a subsequent modelling analysis of climate and vegetation shifts in the highlands of Oaxaca for the aforementioned *Geotrupes* and *Phanaeus* species.

## Supplementary Material

XML Treatment for Phanaeus (Notiophanaeus) dionysius

XML Treatment for Phanaeus (Notiophanaeus) malyi

XML Treatment for Phanaeus (Notiophanaeus) bravoensis

XML Treatment for Phanaeus (Notiophanaeus) halffterorum

XML Treatment for Phanaeus (Notiophanaeus) huichol

XML Treatment for Geotrupes (Onthotrupes) pecki

XML Treatment for Geotrupes (Onthotrupes) viridiobscurus

## Supplementary materials

Supplementary material 1 Figure S1 Data type: Figure (PNG file) Explanation note: Pronota close-ups of **a** *Ph. pyrois olsoufieffi* **b** *Ph. malyi*, and **c** *Ph. pyrois funereus*. Scale bar = 5mm. This is an open access article distributed under the terms of the Creative Commons Attribution License (CC BY 4.0), which permits unrestricted use, distribution, and reproduction in any medium, provided the original author and source are credited.Bert Kohlmann, Alfonsina Arriaga-Jiménez, Matthias Rös
