# Supplementary figures and images for: Dung beetle vicariant speciation in the mountains of Oaxaca, Mexico, with a description of a new species of Phanaeus (Coleoptera, Geotrupidae, Scarabaeidae)
Source: Zookeys. 2018 Mar 14;(743):67–93. doi: 10.3897/zookeys.743.23029 (PMC5904386; doi:10.3897/zookeys.743.23029)

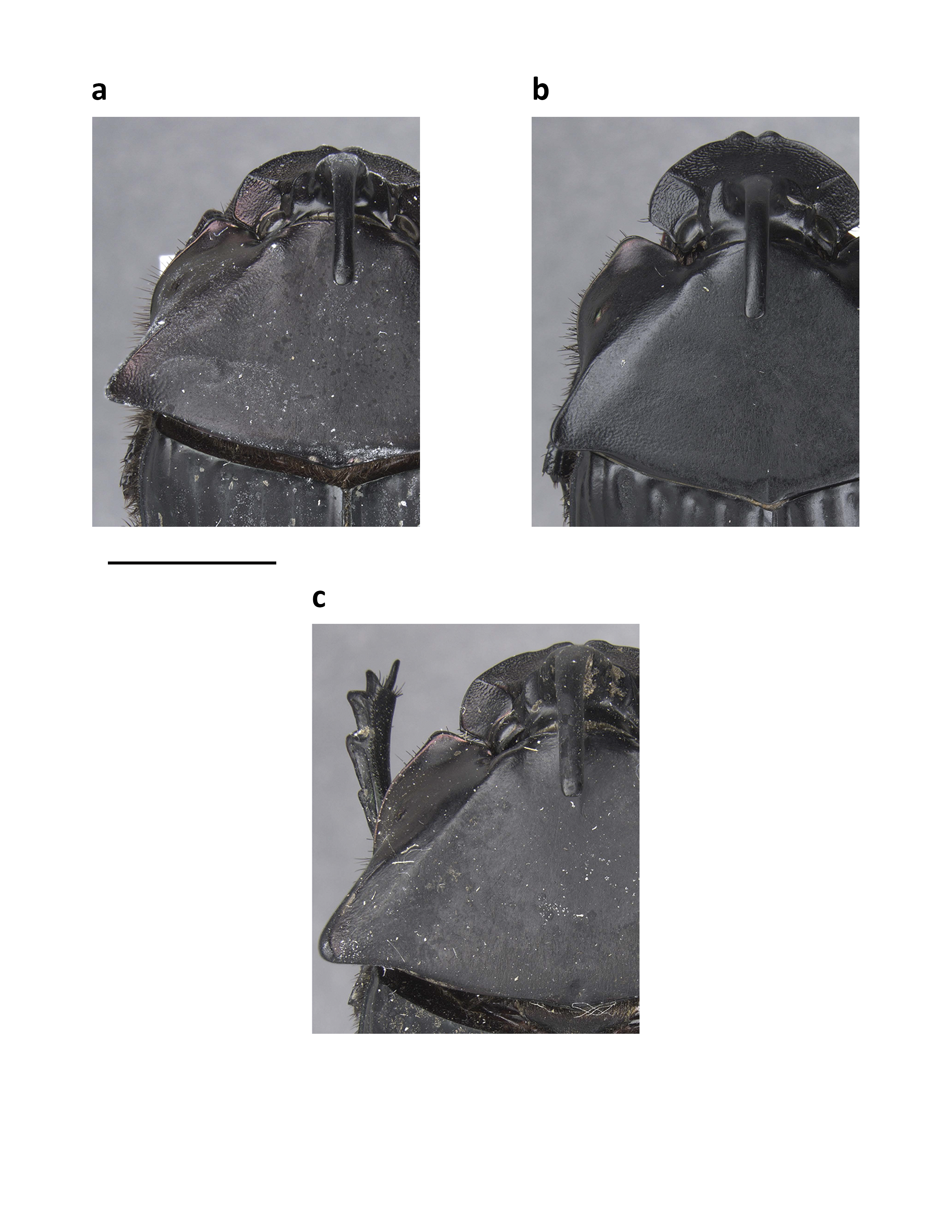

Supplement: Supplementary material 1 — Figure S1 [file zookeys-743-067-s001.png]
